# Formation of Neutral Peptide Aggregates as Studied by Mass‐Selective IR Action Spectroscopy

**DOI:** 10.1002/anie.201902644

**Published:** 2019-06-28

**Authors:** Sjors Bakels, Sebastiaan B. A. Porskamp, Anouk M. Rijs

**Affiliations:** ^1^ Radboud University Institute for Molecules and Materials FELIX Laboratory Toernooiveld 7c 6525 ED Nijmegen The Netherlands

**Keywords:** aggregation, IR spectroscopy, laser desorption, peptides, self-assembly

## Abstract

The spontaneous aggregation of proteins and peptides is widely studied owing to its relation to neurodegenerative diseases. To understand the underlying principles of peptide aggregation, elucidation of structure and structural changes upon their formation is key. This level of detail can be obtained by studying the peptide self‐assembly in the gas phase. Structural characterization of aggregates is mainly done on charged species, as adding charges is an intrinsic part of the technique to bring molecules into the gas phase. Studying neutral peptide aggregates will complement the existing picture. These studies are restricted to dimers due to experimental limitations. Herein, we present advances in laser desorption molecular beam spectroscopy to form neutral peptide aggregates consisting of up to 14 monomeric peptides in the gas phase. The combination of this technique with IR–UV spectroscopy allowed us to select each aggregate by size and subsequently characterize its structure.

Self‐assembly of proteins and peptides into distinct ordered structures has triggered interest in a wide variety of research fields ranging from biology^1^ to the development of smart materials^2^ and life‐like nanosystems.^3^ Amongst these examples, protein and peptide aggregation is mostly studied in relation to human disorders, such as the neurodegenerative diseases Alzheimer's and Parkinson's Disease, where the formation of aggregates into amyloid fibrils is observed.^4^ Multiple techniques have been used to provide insight into the aggregation process, such as transmission electron microscopy,^5^ cryo‐EM,^6^ circular dichroism,^7^ NMR spectroscopy,^8^ and FTIR spectroscopy.^9^ These methods were able to reveal the structures of full‐grown fibrils, but lack the ability to determine the structures forming in the toxic early stage, such as dimers, trimers, and small oligomers, owing to a high degree of complexity and heterogeneity.

Mass spectrometry coupled with techniques such as infrared spectroscopy and/or ion mobility can shed light on the structure of these oligomers.^10^ Electrospray ionization is commonly used to bring these aggregates as charged species into the mass spectrometer. The initial steps of aggregate formation have been probed by mass spectrometry combined with IR spectroscopy and ion mobility, focusing on the amyloid‐prone peptides VEALYL (insulin) and NFGAIL (human islet amyloid polypeptide).^11^ An increase in β‐sheet character in the IR signatures, indicative of the formation of fibrils, was observed in the more extended oligomers. Charge plays an important role, since it can induce unfolding and consequently alter the structure of the peptide. Studying neutral peptides will bring insight into non‐charge‐driven structural preferences upon aggregation. However, the formation of aggregates of neutral peptides is not straightforward. There are only a handful examples of studies on peptide dimers, in which either a heatable source or laser desorption was used to bring them into the gas phase. The groups of Gerhards and Rijs/Gaigeot have studied the structure of neutral (Ac‐Phe‐OMe)_2_ using IR–UV spectroscopy.^12^ They concluded that these peptide dimers are oriented in an antiparallel β‐sheet structure. Snoek and co‐workers investigated a larger peptide dimer: the neutral and charged amyloidogenic peptide sequence VQIVYK.^13^ They showed that the charge on the lysine prevents the formation of extended peptide backbone segments as observed in amyloid fibrils. In contrast, the studied neutral dimer did form a β‐sheet structure in the gas phase. Recently, we studied the competition between intra‐ and intermolecular interactions upon dimer formation of alanine‐containing peptides.^14^

Here, for the first time, we demonstrate the formation of stable higher‐order clusters of neutral peptides in the gas phase using laser desorption (the experiment is described in the Experimental Section and Supporting Information). The peptide used in our experiments is the capped dipeptide Ac‐Ala‐Ala‐OBn (BioMatik, >95 % purity, molecular weight of 292.31 amu). Its mass spectrum (Figure 1) was recorded at the resonant ionization wavelength of the trimer (37 460 cm^−1^) and shows a progression of peaks with increments of *m*/*z* 292 associated with the growth of (Ac‐Ala‐Ala‐OBn)_*n*=1–14_ clusters. All aggregates have their absorption maximum around the trimer wavelength, except for the slightly blue‐shifted dimer (see the Supporting Information). Therefore, it is possible to measure the IR spectra of all clusters simultaneously. The formation and detection of these large aggregates is achieved by adjusting multiple experimental parameters, such as sample preparation (ratio sample/carbon black), gas pulse (density, length), desorption and excitation laser power and delay, excitation laser frequency, and time‐of‐flight settings. The most dominant effect is obtained in the desorption step, resulting in the enhancement of aggregate formation. The desorption laser (Figure 2 a, picture not drawn to scale) is mildly focused on a graphite sample bar, resulting in a beam diameter of about 1 mm and power of 1 mJ/pulse. The sample bar can be varied in height with respect to the axis of the molecular beam (blue arrow). The opening of the nozzle (grey) has a diameter of 0.5 mm (black arrow). After desorption, which takes place inside the gas pulse, the molecules are seeded in a supersonic argon expansion.

**Figure 1:**
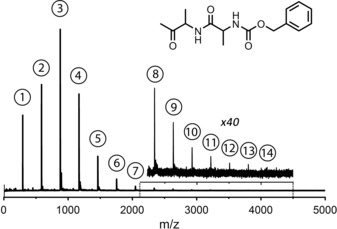
Mass spectrum of the studied peptide Ac‐Ala‐Ala‐OBn (structure shown) as obtained at the trimer resonant ionization wavelength of 37 460 cm^−1^. The numbers indicate the amount of monomeric peptide units involved in each aggregate.

**Figure 2:**
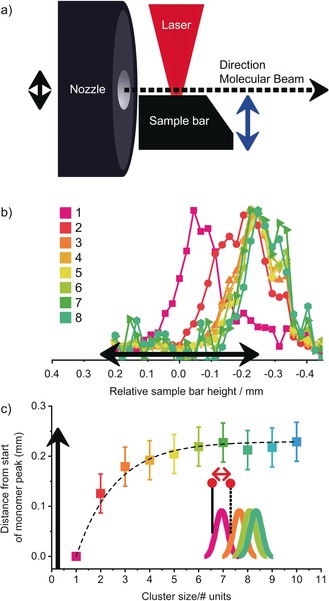
a) Desorption setup. b) Normalized signal of multimers of Ac‐Ala‐Ala‐OBn with respect to the position of the sample bar, where zero marks the nozzle centre. c) Difference between the start (at FWHM) of each multimer with respect to the start at FWHM of the monomer. The black arrow indicates the nozzle diameter.

The position of the sample with respect to the nozzle opening is key for optimal performance.^15^ Changing the height of the sample bar changed the signal as shown in Figure 2 b. The normalized signal per multimer was monitored as function of the vertical distance of the desorption surface relative to the center of the nozzle. The optimal height for monomers was slightly below the center of the molecular beam axis, such that the opening of the nozzle was partly blocked.^16^ Lowering the sample bar (negative *x*‐axis values), that is, increasing the distance between the sample bar and the center of the molecular beam, significantly increased the amount of observed multimers. The multimer signal appeared to continue for about 0.1 mm below the nozzle orifice, whereas the monomer signal vanished below the nozzle opening (Figure 2 b; see also the Supporting Information). The observed effects are not mass‐dependent, that is, monomers of any mass up to *m*/*z* 1500 were typically found just below the center of the nozzle orifice. In contrast, clusters were always found near the lower edge of the nozzle opening, as can be seen in Figure 2 c, which shows the difference between the rising edge at the full width at half maximum (FWHM) of each aggregate with respect to the monomer (see inset). Each point represents the average of multiple measurements taken at three different UV excitation wavelengths. The zero position coincides with the center and 0.25 mm with the lower edge of the nozzle opening.

The longer pathway for the multimers allows for more collisions with present peptide molecules and argon atoms; molecules have more time to cool down and spend more time in a denser environment undergoing collisions required for aggregate formation. This effect becomes more pronounced until the sample bar is moved too far away from the molecular beam path, resulting in a sudden drop in the signal. The high efficiency of the cooling and clustering process explains why all monomers that are present when partly blocking the nozzle are completely consumed in the aggregation process when the sample bar is lowered.

The mass‐selective IR spectra of the Ac‐Ala‐Ala‐OBn clusters with *n*=2–7 were measured in the fingerprint region from 1000 to 1800 cm^−1^ (Figure 3 a). The region between 1600 and 1800 cm^−1^ comprises the C=O stretching vibrations and shows two distinctive peaks. The smaller peak above 1700 cm^−1^ results from the C=O group of the ester moiety present at the OBn‐cap, whereas the peak between 1600 and 1700 cm^−1^ originates from the peptide C=O groups (amide I). Every peptide monomer has one ester C=O and two peptide C=O groups. The amide II band (NH bend) appears as a broad feature located between 1490 and 1570 cm^−1^. Other distinctive features include the large bands around 1200 cm^−1^, originating from backbone and amide III motions. Figure 3 b,c displays the normalized IR spectra for aggregates with *n*=2–7 in the amide I and II region, respectively. These regions are sensitive to hydrogen bonding, thereby providing structural details on intra‐ and intermolecular interactions.

**Figure 3:**
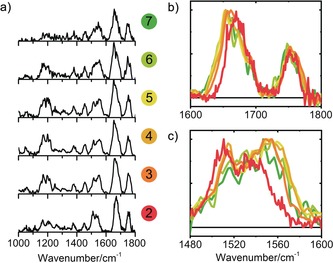
a) Mass‐selective IR action spectra of Ac‐Ala‐Ala‐OBn aggregates. The number of monomeric units in a multimer is designated on the right. b,c) Spectra of clusters with *n*=2–7 (2 red, 3 dark orange, 4 orange, 5 yellow, 6 light green, and 7 green) in the amide I (C=O stretch) region (b) and amide II (NH bend) region (c).

Quantum chemical calculations were performed to structurally assign the experimental IR spectra. For the dimer, eight structural families were determined based on their hydrogen‐bond patterns (see the Supporting Information). The calculated IR spectrum of the lowest energy structure shows the best agreement with the experimental spectrum. The dimer was therefore assigned to this structure, composed of a parallel β‐sheet (Figure 4 a). It is formed by two intermolecular hydrogen bonds, whereby the backbones of the two peptides are aligned in a parallel orientation. The ester C=O groups are not involved in any hydrogen bonding. The individual peptides present in the parallel β‐sheet dimer retain their monomeric structures: the linear and the γ‐turn conformer are both present in the dimer, in which their weak intramolecular hydrogen bonds (C5 hydrogen bond and NH–π bond) are replaced by stronger intermolecular hydrogen bonds.^14^, ^17^ The calculated IR spectrum of an antiparallel dimer is also compared with the recorded IR spectrum in Figure 4 a. This structure, like the parallel dimer, has no ester C=O groups involved in hydrogen bonding; however, was discarded as a result of a mismatch in the amide II region together with its high energy.

**Figure 4:**
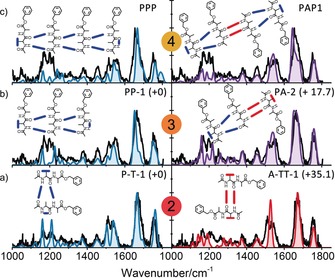
Experimental (black) and calculated IR spectra (coloured) of the a) dimer, b) trimer, and c) tetramer. The spectra of the all‐parallel C12 families are shown on the left in blue; the best‐matching spectra for combinations with antiparallel β‐sheets are shown on the right. The relative zero‐point corrected energy for each aggregate is shown in the right‐hand top corner together with the name given to the aggregate. Tetramer energies are not shown as they are calculated with different basis sets (see Table SI.1 in the Supporting Information).

In the IR spectra of the aggregates with *n*>2, the peak between 1700 and 1800 cm^−1^, originating from the ester C=O moiety, did not significantly change position or width. This indicates that for the higher‐order clusters the ester C=O groups are also not involved in any hydrogen‐bonding interaction. In contrast, the position of the amide I (Figure 3 b; see also Figure SI.7B in the Supporting Information) and amide II bands (Figure 3 c) shifted to the red and blue, respectively, indicative of an increased number of, and hence stronger, hydrogen bonds.^18^ These shifts became smaller as higher‐order clusters were formed.

Calculations showed that the low‐energy structures of the trimer were exclusively β‐sheet‐type structures. Other structural families (globular) were at least 25 kJ mol^−1^, but often over 40 kJ mol^−1^ higher in energy. The experimental spectrum only matches to structures in which all three monomers are attached through β‐sheet intermolecular hydrogen bonds (see the Supporting Information). In particular, conformers which arise from the attachment of the third peptide to the previously assigned dimeric structure P‐T‐1 are in good agreement. The third peptide can aggregate on both sides of the dimer: at the C7 γ‐turn intramolecularly or weaker C5 intramolecularly hydrogen bonded side. On the basis of energetics and spatial orientation, it is expected that the weaker C5 hydrogen bond is broken to favor the formation of strong intermolecular β‐sheet interactions.^14^ Two β‐sheet‐containing structures remain possible: an all‐parallel structure and a structure in which the third monomer is attached in an antiparallel fashion to the dimer. The added monomer peptide adopts either the observed linear conformer (for the all‐parallel structure; Figure 4 b, left) or the γ‐turn conformer for the antiparallel structure (Figure 4 b, right). The antiparallel structure shows a better overlap in the region around 1200 cm^−1^; however, the all‐parallel structure has considerably (17 kJ mol^−1^) lower energy and reproduces the double peak in the amide II region better. Overall, the structure of the trimer can be confidently assigned to an all‐β‐sheet structure, in which the peptides are stacked on top of each other. Energetics and spectral features, such as the shoulder in the amide I region, point to the presence of parallel β‐sheets.

The theoretical IR spectra of all‐β‐sheet tetramer structures show good agreement with the experiment (see the Supporting Information). Additionally, the energetics showed that, as was observed for the dimer and the trimer, the all‐parallel structure has the lowest energy. The spectra of two possible structures are shown in Figure 4 c: an all‐parallel conformer (6‐31G* basis set) and a mixed (anti)parallel low‐energy conformer (6–31+G* basis set). Both conformers can originate from the addition of a monomer to one of the two discussed trimers, or by merging two dimers. Spectrally, the overlap for both structures is good in the amide I region, although the all‐parallel conformer was calculated on a lower level of theory and therefore has a smaller peak around 1800 cm^−1^ (see the Supporting Information). The remaining parts of the spectra show reasonable overlap for both β‐sheet conformers. In line with the dimer and trimer, the tetramer structure has all peptides stacked in β‐sheets on top of each other. On the basis of energetics and structures of the dimer and trimer, the formation of an all‐parallel tetramer is expected.

The observed red and blue shifts of the amide I and II bands, respectively, in the IR spectra continue for the higher‐order clusters (*n*>4). For the assignment of these clusters, we relied on size‐related shifts in the spectrum and comparisons with the smaller aggregates rather than on DFT calculations (see the Supporting Information). A similar trend for aggregation onwards from the tetramer is expected, in which the peptides aggregate into stacked β‐sheet‐containing structures, whereby the weak intramolecular hydrogen bonds are broken to favor stronger intersheet hydrogen bonds. The observed red shift in the amide I region for the larger aggregates continues towards the amide I band of the solid‐state FTIR spectrum of Ac‐Ala‐Ala‐OBn. This spectrum was recorded using a KBr pellet (see Supporting Information) and shows a clear signature at 1629 cm^−1^, corresponding to parallel β‐sheets.^19^ The difference between the condensed phase and the gas phase is ascribed to interactions with the environment, in this case the other peptides in the matrix.^20^

In conclusion, we have shown that it is possible to make aggregates of neutral peptides in the gas phase by allowing the laser‐desorbed peptides more time to cool down in a denser environment. The amount of neutral aggregated peptides (*n*=14) is unprecedented, as peptide dimers were the highest‐order clusters created previously. IR–UV ion dip spectroscopy and quantum chemical calculations allowed us to assign the structures of the aggregates (*n*=2–4) to predominantly parallel β‐sheets, with the peptides stacked on top of each other. Higher‐order clusters (up to *n*=7) showed a shift in the amide I band towards known β‐sheet signatures observed in the solid phase, thus indicating that the structural preferences observed in the gas phase are related to those in the bulk. The presented results pave the way to complementary studies on neutral biologically active peptides, such as the hydrophobic amyloidogenic peptides.

## Experimental Section

The neutral peptide aggregates were formed in a laser desorption molecular beam time‐of‐flight mass spectrometer.^15^ Conformational and mass‐selective IR spectra were recorded by IR–UV ion dip spectroscopy using the free‐electron laser FELIX. The amber force field and Gaussian 09 were used for our calculations. See the Supporting Information for details of the experiment.

## Conflict of interest

The authors declare no conflict of interest.

## Supporting information

As a service to our authors and readers, this journal provides supporting information supplied by the authors. Such materials are peer reviewed and may be re‐organized for online delivery, but are not copy‐edited or typeset. Technical support issues arising from supporting information (other than missing files) should be addressed to the authors.

Supplementary
