# Supplementary material for: Formation of Neutral Peptide Aggregates as Studied by Mass‐Selective IR Action Spectroscopy
Source: Angew Chem Int Ed Engl. 2019 Jun 28;58(31):10537–41. doi: 10.1002/anie.201902644 (PMC6772166; doi:10.1002/anie.201902644)
Supplement: Supplementary file 1 — Supplementary [file ANIE-58-10537-s001.pdf]

Angewandte Chemie
Eine Zeitschrift der Gesellschaft Deutscher Chemiker

Supporting Information

Formation of Neutral Peptide Aggregates as Studied by Mass-Selective IR Action Spectroscopy

*Sjors Bakels, Sebastiaan B. A. Porskamp, and Anouk M. Rijs**

anie_201902644_sm_miscellaneous_information.pdf

Author Contributions

A.R. Conceptualization: Lead; Data curation: Supporting; Formal analysis: Equal; Methodology: Equal; Supervision: Lead; Writing—Original Draft: Equal; Writing—Review & Editing: Lead
S.B. Data curation: Lead; Formal analysis: Lead; Investigation: Lead; Writing—Original Draft: Equal
S.P. Data curation: Supporting; Formal analysis: Supporting; Investigation: Supporting; Writing—Original Draft: Supporting.

List

1. Experimental details
2. REMPI of monomer and aggregates of Ac-Ala-Ala-OBn

   - FIG.SI.2: UV excitation spectra of $(\text{Ac-Ala-Ala-OBn})_{n=1-5}$
3. Influence of sample bar position on aggregation

   - FIG.SI.3: Sample bar height plots
4. Structural assignment of the dimer of Ac-Ala-Ala-OBn

   - FIG.SI.4A: Dimer – energies

   - FIG.SI.4B: Dimer – IR spectra
5. Structural assignment of the trimer of Ac-Ala-Ala-OBn

   - FIG.SI.5A: Trimer – energies

   - FIG.SI.5B: Trimer – IR spectra
6. Structural assignment of the tetramer of Ac-Ala-Ala-OBn

   - FIG SI.6A: Tetramer – IR spectra at 6-31G*

   - FIG.SI.6B: Tetramer – Basis set differences

   - Table.SI.1: Tetramer - Energies

   - FIG.SI.6C: Tetramer – IR spectra at 6-31+G*
7. FTIR data of Ac-Ala-Ala-OBn and C=O peak positions

   - FIG.SI.7A: FTIR spectrum of solid-state Ac-Ala-Ala-OBn

   - FIG.SI.7B: Fitted C=O peak positions
8. References in ESI

1. Experimental details

Experimental set-up

The experimental set-up as it is used has been extensively described elsewhere.[1] In short, the molecules of interest are mixed with carbon black, and subsequently deposited on a graphite sample bar. The sample bar is then irradiated with about 1 mJ of 1064 nm light, resulting in desorption of the sample molecules. These are then entrained in a supersonic expansion of argon atoms and cooled down (10 Hz pulsed valve (Jordan)). The position of the sample bar with respect to the nozzle opening can be varied by moving the sample bar stage. The entrained molecules pass a skimmer, and subsequently interact with laser light. UV laser light (1 mJ/pulse) is provided by a Nd:YAG pumped dye laser (Radiant Dyes/LiopTec), and is used in a 1+1 REMPI process, where it excites and ionizes the molecules via a resonant transition state. To obtain infrared spectra, the molecules are irradiated by a pulse of infrared laser light (~25 mJ/pulse, ~10 µs duration), provided by the Free Electron Laser FELIX, prior to UV excitation.[2] The ion signal from the UV laser is monitored and shows dips if IR resonances are excited that share a common ground state level with the UV transition. On/off measurements are performed, to correct for fluctuations in the signal, by running the experiment at 10 Hz, and FELIX at 5 Hz.

Quantum chemical calculations

Conformational searches were performed using the amber force field.[3] Molecules were heated up to ~325 Kelvin after which they were cooled down to 0 K. The structures that were found were then clustered on basis of similarity and further optimized. Optimization and frequency calculations were performed in the Gaussian 09 environment, at the B3LYP/6-31+G* level of theory with Grimme's D3 empirical dispersion term.[4, 5] All spectra are scaled with 0.976, but NH bending modes and CH_2 and CH_3 modes between 1400 and 1600 cm^{-1} are scaled with 0.962.[6] For the dimer, an extensive conformational search was performed, starting from several input structures. In total 80 structures were found and optimized. The conformational search on the trimer was complemented with a number of structures arising from the assigned dimer structure, which yielded 28 structures. The size and complexity of the tetramer only allowed the manual design of input structures, which were subsequently optimized and the frequencies calculated using B3LYP-D3 with the 6-31G* or, where possible, the 6-31+G* basis set. The relative zero-point energies can be found in Table SI.1.

2. REMPI of monomer and aggregates of Ac-Ala-Ala-OBn

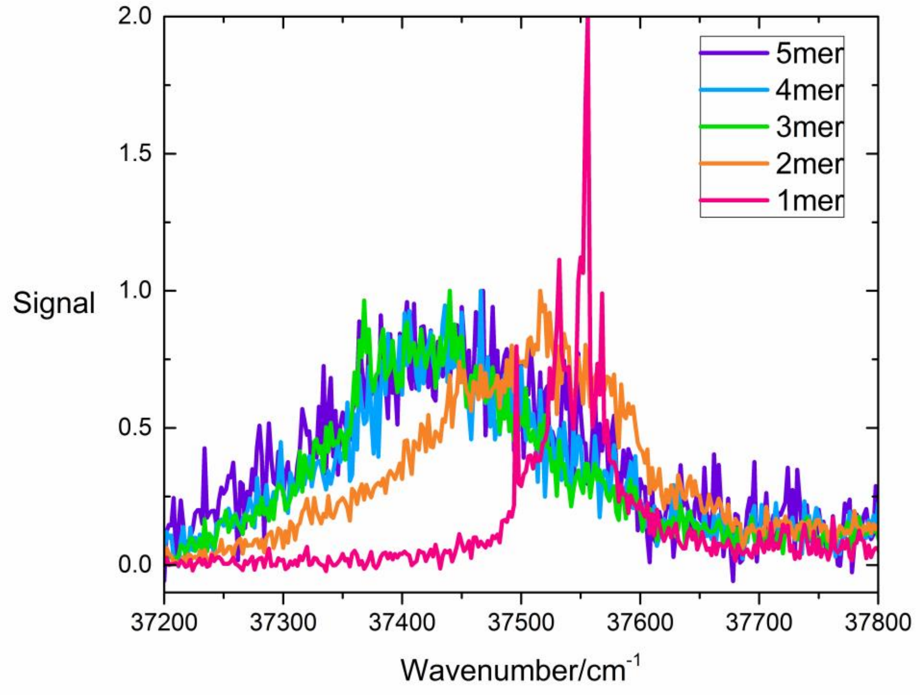

Figure SI.2: Normalized REMPI spectra of the different aggregates of Ac-Ala-Ala-OBn, with n=1-5. The signal of the monomer is multiplied by 2 for clarity.

3. Influence of sample bar position on aggregation

We measured the signal of the mass peak of each aggregate versus the sample bar height perpendicular to the molecular beam axis. From these plots, as shown in Figure 2b (main text) and schematically in Fig.SI.3g, the peaks were examined and the position of the FWHM on both sides and the maximum of the peak were listed. The difference between the position of the FWHM on the rising edge (i.e. close to the center of the nozzle opening) and the three values for each multimer (Fig.SI.3g) were then taken for every multimer. These height measurements were performed using several different excitation wavenumbers, with different amount of measurements per wavenumber, and were all plotted separately (Fig.SI.3a-e) and together (Fig.SI.3f). Measurements on the lower wavenumbers (Fig.SI.3a-c) show that often the end of the monomer is before the start of the dimer, meaning that almost no dimer signal is observed when the monomer is observed. Experiments done closer to the monomer wavelength (Fig.SI.3d), or on resonance with the monomer (Fig.SI.3e) show that this behavior is less extreme, but still clearly present.

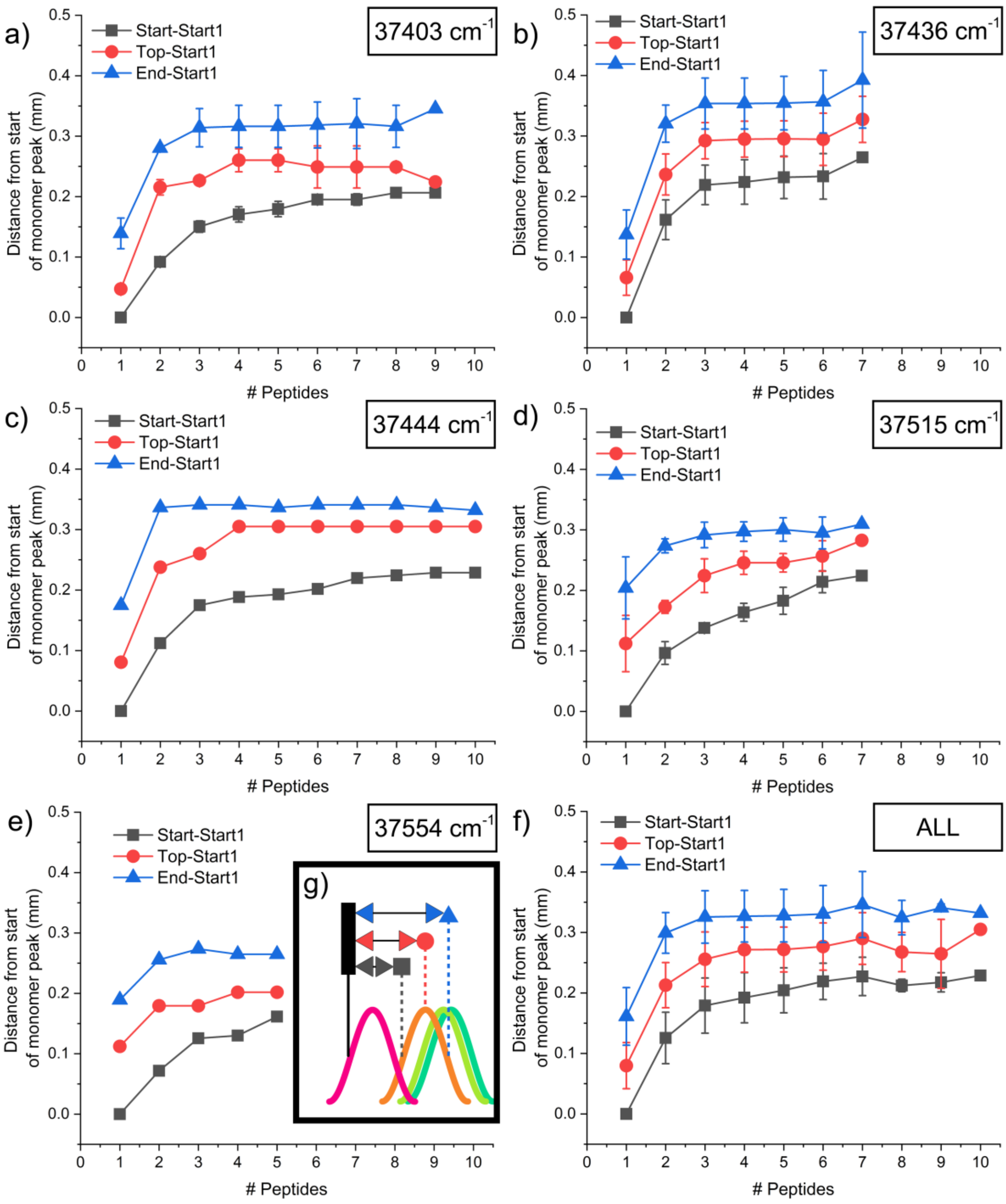

Figure SI.3: a-e) Plots of start (grey), top (red) and end (blue, all at FWHM) of every multimer with respect to the start at FWHM of the monomer peak, taken from height plots of the sample bar versus signal, at different wavelengths; f) All measurements combined, showing the general trend; g) Explanation of the measurements, all peaks are signal versus sample bar height.

4. Structural assignment of the dimer of Ac-Ala-Ala-OBn

The experimental spectrum of the dimer of Ac-Ala-Ala-OBn shows a number of distinctive features. Starting with the amide I region, which consists of two peaks: one above 1700 cm^{-1} and one between 1620 and 1700 cm^{-1}. The peak above 1700 cm^{-1} can be assigned to a stretch vibration of the two C=O groups present in the ester cap at the C-terminus. The peak between 1620-1700 cm^{-1} consists of 4 peptide C=O groups. The peak position of the ester C=O around 1750 cm^{-1} indicates that none of these C=O groups are involved in any sort of hydrogen bonding, and are thus free in the conformer present in our experiment. The peptide C=O peaks show a small shoulder around 1692 cm^{-1}, which is indicative of either a non-hydrogen bonded C=O or a weak intramolecular hydrogen bond such as a C5 interaction. Most of the intensity, however, concentrates around 1668 cm^{-1}, which points to stronger hydrogen bonds, such as intermolecular hydrogen bonds or a C7 intramolecular hydrogen bond. The amide I region indicates thus the presence of strong intermolecular hydrogen bonds between the peptide C=O groups, with the ester C=O groups not involved in any sort of hydrogen bonding.

The amide II region (N-H bending modes) shows two bands, at 1510 cm^{-1} and one around 1535 cm^{-1}. This is indicative of weak and strong hydrogen bonded N-H groups, respectively. The rest of the spectrum shows features at 1450 cm^{-1}, 1375 cm^{-1} (both CH_3, CH_2 and ring motions), 1259 cm^{-1} (amide III motions), broader bands around 1205 cm^{-1}, a large peak at 1165 cm^{-1} (both backbone motions involving the OBn cap) and smaller ones at 1109 cm^{-1} and 1047 cm^{-1} (both backbone motions involving the alanine side groups).

Calculations on the dimer were done using Gaussian 09 with the B3LYP-D3 6-31+G* level of theory. Multiple rounds of molecular dynamics (MD) simulations were performed in order to sample the complete potential energy landscape. Here, starting from an input structure, the molecule is heated to temperatures above 300 K and is subsequently frozen into an energetic minimum. This is repeated 500 times, after which the 500 obtained structures are clustered together on basis of similarities. However, the temperature chosen to cross the energetic barriers and consequently sample the full landscape, is restricted for dimers. When the temperature is above 350 Kelvin the dimer breaks its intermolecular hydrogen bonds and the monomers fly away from each other. However, low temperatures make it unlikely that the

full potential landscape is sampled, which is why multiple rounds were started, all from completely different input structures. For the dimer of Ac-Ala-Ala-OBn 80 structures were found, which were categorized into 8 structural families. These are shown in Fig.SI.4A(b). The families are determined on basis of their hydrogen bond pattern and can be categorized into three main groups. When the dimer has two intermolecular hydrogen bonds it can form the structure in a so-called beta-sheet type structure, or in a globular folded structure.

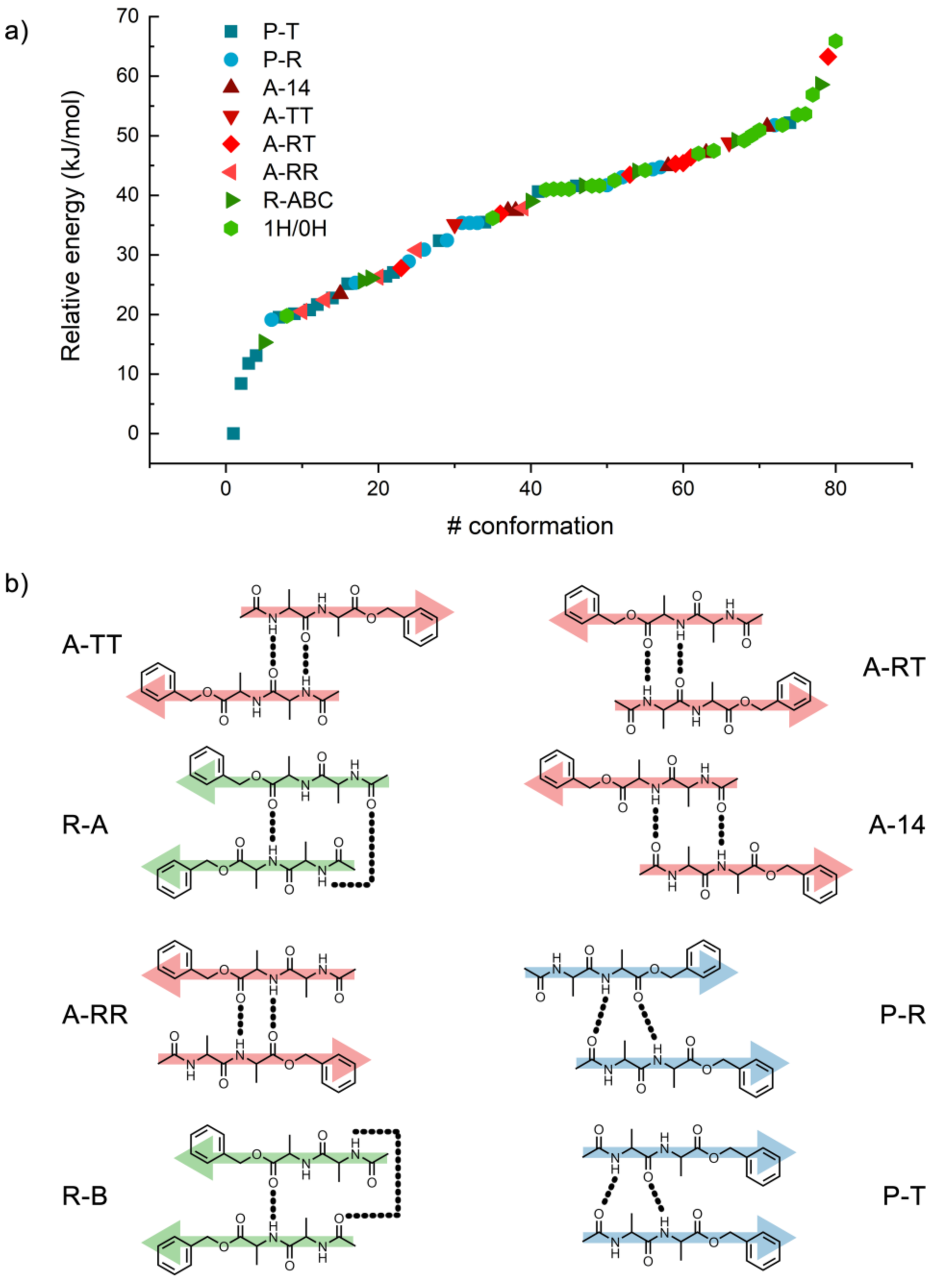

Figure SI.4A: a) Relative zero-point corrected energies for the dimer of Ac-Ala-Ala-OBn. The different colors represent the families as shown below; b) All 8 structural families used to categorize the calculated dimers. In blue the parallel (P) beta sheet structures, in red anti-parallel (A) structures, and in green the other (R) structures.

In the beta-sheet case the backbones of the two monomeric units can be aligned in a parallel or anti-parallel way to each other, and these two form the first two groups. The third group contains all other structures. The beta-sheet structures can form their parallel or anti-parallel beta sheets using different atoms in the molecule, and these differences form the sub-categorization. The relative zero-point corrected energies of the calculated structures are shown in Figure SI.4A(a), where the color code represent the different families. Up to around 37 kJ/mol the structures are almost exclusively parallel beta-sheets. The calculated spectra of the lowest energy structures of each family are plotted in Fig.SI.4B, along with their relative zero-point corrected energies. Mode dependent scaling factors were used for the dimers: The N-H bending modes in the amide II and the CH_2 and CH_3 modes between 1360 and 1480 cm^{-1} have been scaled by 0.962, while the rest of the spectrum is scaled by 0.976.

Here, only the P-T, A-14 and A-TT structures show similar separations between the two peaks in the amide I region, and all have, accordingly to above findings, no hydrogen bonded ester C=O groups, and strongly hydrogen bonded peptide C=O groups. There are differences however: Where the P-T-1 spectrum has an almost perfect match with the experiment, the A-14-1 spectrum shows two clearly separated peaks in the peptide C=O region, whereas the experimental spectrum shows 1 peak with a small shoulder. The A-TT-1 spectrum, which is the highest energy structure of all families, does not show this shoulder. Moreover, in the amide II region A-TT-1 only shows 1 peak around 1520 cm^{-1} instead of the two bands. Conformer A-14-1 shows two peaks, but they are separated too much, and P-T-1 shows, again, a very good match. The rest of the spectrum also favors the P-T-1 structure, but less convincing as in the amide I and II region. It accounts for all the observed peaks, whereas the other structures do not. This best overlap with experiment, together with the, by far, lowest energy in an extensive conformational search, allows us to confidently assign the structure of the dimer of Ac-Ala-Ala-OBn to this parallel beta-sheet structure.

The structure, named P-T-1, consists of two structurally different monomer peptides: One monomer conformer with a linear structure, with a C5 hydrogen bond, and the other with a γ-turn (C7 hydrogen bond).[6] This is consistent with the monomers found to be present in the experiment, as was previously determined.[6, 7] It appears that the dimer is built up of the monomers, and thereby breaking the weakest intramolecular hydrogen bonds to favour the

strong intermolecular hydrogen bonds. The γ-turn monomer is of particular interest, since the C7 hydrogen bond orients the NH and C=O that are positioned between the hydrogen bonds (pink in Fig.SI.4B, P-T-1) away from each other, thereby creating the perfect angle to form a parallel C12 beta sheet.

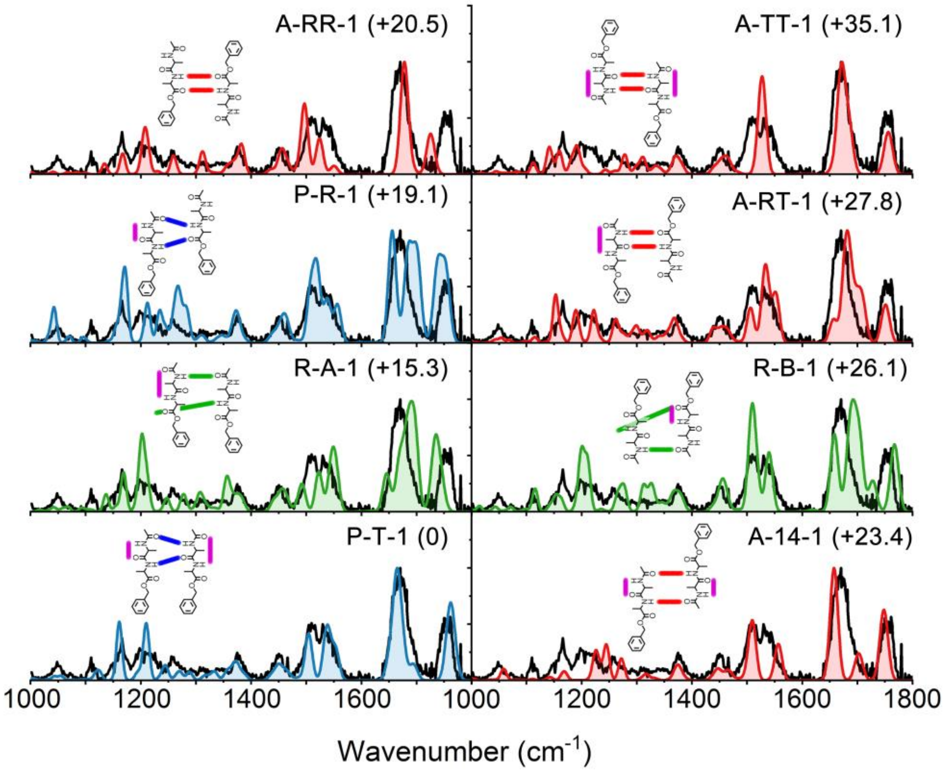

Figure SI.4B: Infrared spectra of the lowest energy structures per family of the dimers of Ac-Ala-Ala-OBn in color, compared to the experimental spectrum in black in energetic order, between brackets is the relative zero-point corrected energy in kJ/mol. The names correspond to the structures as shown in Fig.SI.4A, with the -1 behind the names indicating it is the lowest energy structure of this particular structural family.

Concluding: An extensive conformational search was performed, which resulted in about 80 structures. These were categorized on basis of their hydrogen bonding pattern, and compared to the experimental spectrum. The experimental spectrum can be assigned to the lowest energy structure, which shows the best overlap with experiment. This structure constitutes a parallel intermolecular hydrogen bond between the two peptide units, where two intermolecular hydrogen bonds enclose a ring of 12 atoms. The ester C=O groups are not involved in any hydrogen bonding. The dimer conformer is formed from the two present monomer conformers as was observed previously. The weak intramolecular hydrogen bonds are broken to favor the strong intermolecular hydrogen bonds, thereby maintaining their respective major structural properties (i.e. linear and γ-turn).

5. Trimer assignment

The assignment of the trimer was done in a similar fashion as the dimer, however it appeared to be computationally too demanding. The performed conformational searches were not able to sample the full landscape, and therefore we included specific designed input structures. Following the results from the dimer, which resulted from the two monomer conformers, we took the assigned structure of the dimer and added an extra monomer. This resulted into three possible all beta-sheet structures, as shown in Fig.SI.5B: *i.e.* all parallel conformers (PP), and conformers where the addition of the third monomer resulted in an anti-parallel structure (PA). We also calculated the IR spectrum of an all anti-parallel structure, named AA, for completeness. In Fig.SI.5A the relative energies of all calculated structures are shown, both from the conformational search as well as the above-mentioned structures. The colored graph shows that the low energy structures mainly consist of structures which, partly or completely, contain parallel beta-sheets. The first not parallel structure is over 20 kJ/mol higher than the lowest energy structure.

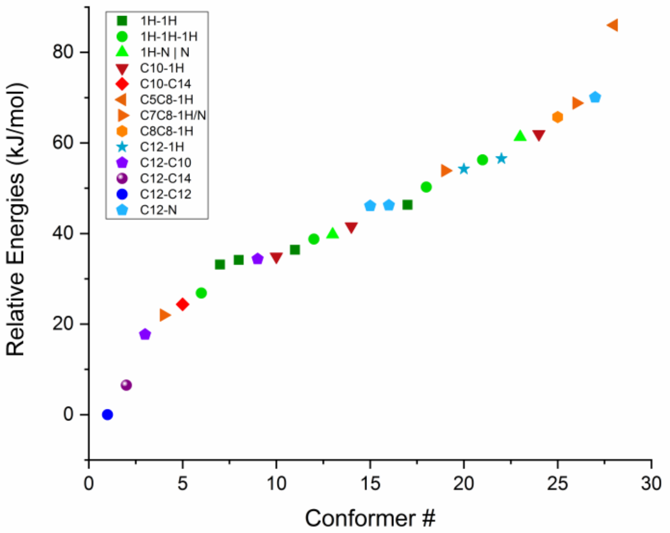

Figure SI.5A: Relative zero-point corrected energies of all calculated conformers of the trimer of Ac-Ala-Ala-OBn and their respective hydrogen bonding patterns: Singly and non-hydrogen bonded complexes in green, anti-parallel hydrogen bonded structures in red, parallel hydrogen bonded structures in blue, partly parallel and partly anti-parallel hydrogen bonded structures in purple, and other hydrogen bonding patterns in orange. All structures are calculated on the B3LYP-D3/6-31+G* level of theory. C10/12/14 means a beta sheet enclosed by that amount of atoms; 1H meaning a single hydrogen bond between two structures, and C5C8 and others mean a ring enclosed by 5+8 atoms.

The experimental spectrum of the trimer of Ac-Ala-Ala-OBn shows similar features as the dimer, but with some differences. The peak above 1700 cm^{-1} originating from the ester C=O groups appear at the same position as was observed for the dimer, indicating that also in the trimer the ester C=O groups are not involved in any hydrogen bonding. The amide I peak however, shows a broadening towards lower wavenumbers, meaning more strongly hydrogen bonded peptide C=O groups are present. The amide II region shows the largest difference, with a slight blue-shift of the low energy side of the band at 1500 cm^{-1}, and a clear broadening and shift of the peak from 1530 cm^{-1} to 1555 cm^{-1}. This confirms the presence of stronger hydrogen bonds. The other main difference from the dimer spectra is the intensity of the peaks around 1200 cm^{-1}, originating from backbone motions, including $C_{\alpha}H$, which are increased with respect to the dimer.

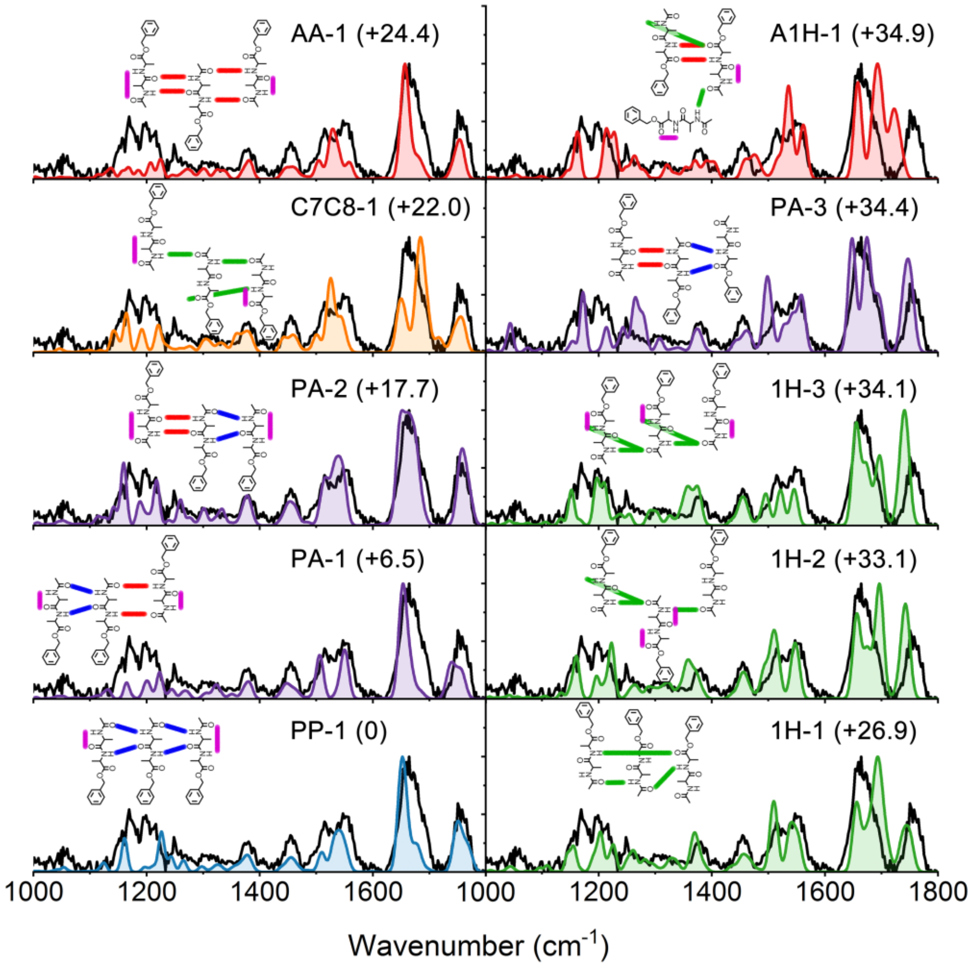

Figure SI.5B: Calculated infrared spectra of the ten lowest energy trimers in color, compared to the experimental spectrum in black in energetic order. In the inset their respective structures are drawn, with red hydrogen bonds indicating an anti-parallel beta sheet and blue a parallel one. Green and pink hydrogen bonds are other intermolecular and intramolecular hydrogen bonds, respectively. The names of the structures indicate which hydrogen bond types are present (P=parallel, A=anti-parallel).

The calculated IR spectra are plotted in Fig.SI.5B in color, on top of the experimental IR spectrum in black. The amide I already gives away the most important conclusion: Only the fully beta-sheet structures, so where all three monomers are attached to each other in a stacked manner, thereby creating beta-sheets, show good agreement between experiment and theory, i.e. structures PP-1, PA-1, PA-2, AA-1 and PA-3. The amide II region allows us to further reduce this by excluding the all anti-parallel structure AA-1, which shows a strong peak at 1528 cm^{-1} instead of two bands with their maximum intensities at 1516 and 1550 cm^{-1}. Also PA-3 can be excluded based on this region. The rest of the spectrum unfortunately does not enable us to draw conclusive assignments on the structure. Since only one all parallel structure is calculated, it is highly likely that another low energy structure, similar to these but slightly different, can be found. Continuing the growth mechanism as observed for the dimers, where one monomer conformer was added to the other present conformer, it is expected that the trimer results from the addition of one of the observed monomers on to the dimer conformer (P-T-1). Therefore, it is not likely to form the PA-1 conformer since this requires the breakage of a strong C7 intramolecular hydrogen bond. PP-1 is more likely to be the observed trimer structure, since a linear monomer can attach on the C5 side of the linear monomer. The same holds for the last of the three, PA-2.

Concluding: The size of the trimer makes it computationally more demanding. Therefore only a small conformational search has been performed, followed by an optimization step and frequency calculation together with a number of structures arising from the assigned dimeric structure. The energetics show that the beta-sheet structures are the lowest in energy, with all parallel structure as the lowest conformer. The experimental spectrum only matches to structures where all three monomers are attached via beta-sheets, i.e. specifically to the conformers which arise from the before assigned dimeric structure P-T-1. This structure has two sides on which a third peptide can be added: a C7 intramolecular hydrogen bonded side and a weaker C5 intramolecular hydrogen bonded side. It is expected that this last hydrogen bond is favourable to be broken in favor of stronger intermolecular hydrogen bonds. Two structures remain possible: an all-parallel structure and a structure where the third monomer is attached anti-parallel to the dimer. The added monomer conformer is most probably different in the two cases: Linear in the first case and either linear or γ-turn in the second case.

6. Tetramer assignment

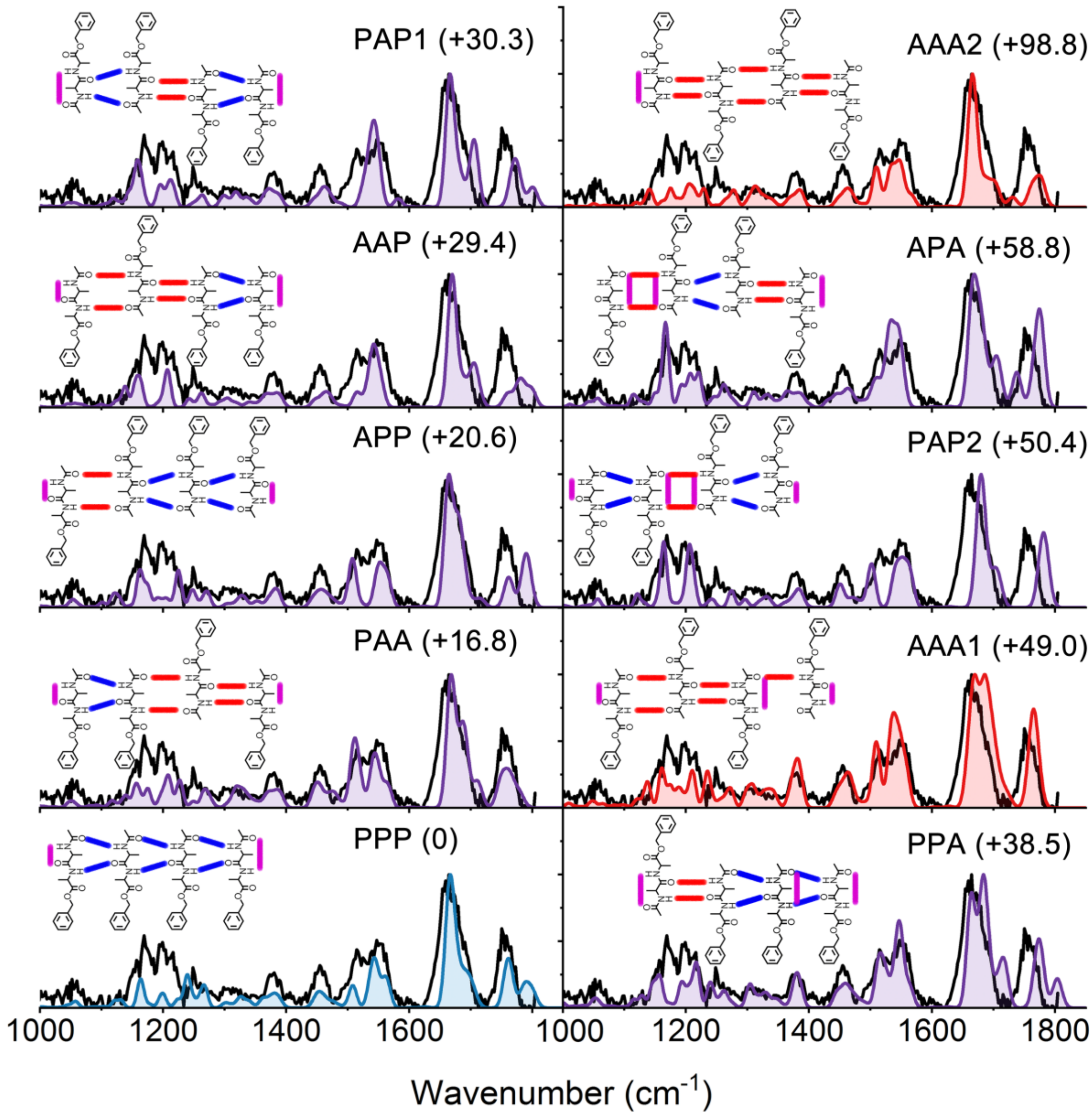

Figure SI.6A: Infrared spectra of ten possible beta sheet stacked tetramers in color, at the B3LYP-D3/6-31G* level of theory, compared to the experimental spectrum in black. In the insets their respective structures, with red hydrogen bonds indicating an anti-parallel beta sheet and blue a parallel one. The names of the structures indicate which hydrogen bond types are present (P=parallel, A=anti-parallel). The relative zero-point corrected energies are put between brackets in kJ/mol. The pink hydrogen bonds indicate intramolecular hydrogen bonds.

The geometry optimizations and frequency calculations of the tetramers, and also higher order clusters, are very time consuming. Since the calculations, when executed using similar basis sets and functional, have an exponential relationship with size of the molecule, one can expect the higher order clusters to be very costly to calculate. For the calculated tetramer spectra in this section, computational times of around 5000 hours were necessary (for the

dimer this was around 200 hours). The fact that the conformational space also increases with size adds to the complexity of the study on higher order aggregates.

The proposed growth mechanism means that the tetramer is either formed by the addition of a monomer to the trimer, or by the addition of a dimer to another dimer. Both options can have different end products. Therefore all possible structures where the four peptides are stacked on top of each other in beta-sheets were calculated. The results using a lower basis set, 6-31G*, are shown in Fig.SI.6A. Here, the all parallel structure (blue), all anti-parallel structures (red), and the combinations (purple) are shown in energetic order. For 5 of these 10 structures we were able to calculate the frequencies on a higher level of theory, using 6-31+G* as basis set. Spectrally, the most important changes between these two basis sets are in the amide I region, where all the peaks shift to the red, and the peak above 1700 cm^{-1}, where the double peak resulted in a single peak. The rest of the spectrum only showed minor changes when changing basis set. An example is shown in Fig.SI.6B.

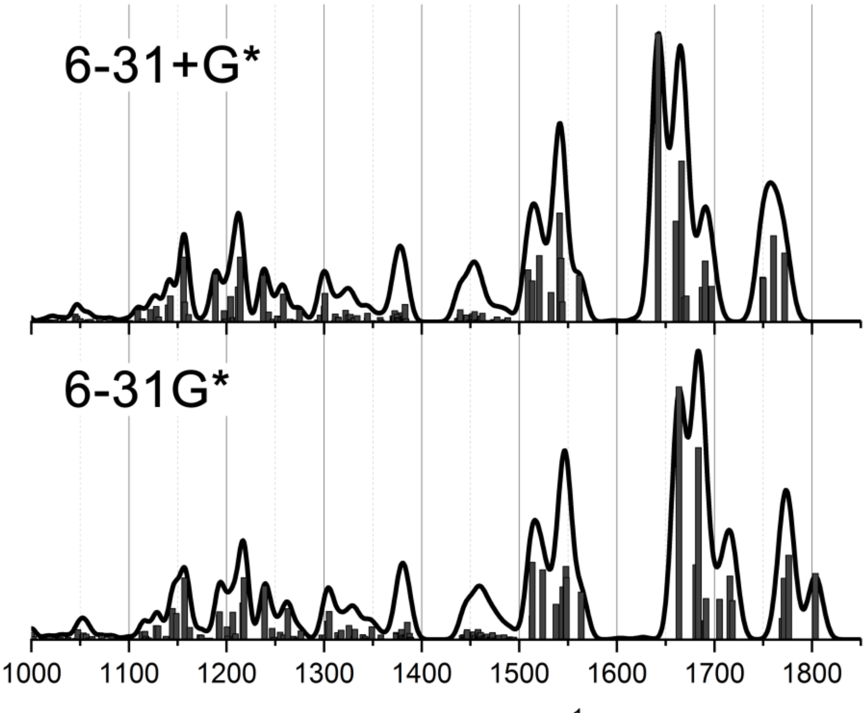

Figure SI.6B: Comparison between structure PPA calculated with both basis sets: Bottom panel 6-31G* and upper panel 6-31+G*. The main differences can be found between 1600 and 1850 cm^{-1}.

The calculated spectra of these 5 structures are shown in Fig.SI.6C. The energetics are shown in Table SI.1, where they maintain more or less the same energetic order when changing basis set. It is therefore not unlikely that the lowest energy structure at the lower basis set, PPP, is

also the lowest energy structure at the higher basis set. By not taking the zero-point energy into account actually gives PPP as the lowest energy structure there. This structure is optimized but not frequency calculated using the higher basis set, the other four have not been optimized.

Structures that can arise from addition of a monomer to the all parallel trimer PP-1 are only the tetramers PPP and PPA. APP is also possible but unlikely since it involves the breakage of a C7 hydrogen bond. The higher energy trimer conformer PA-2 is also unlikely to be present as it requires breaking of a C7 intramolecular hydrogen bond on either side to create a tetramer. The third trimer that was suggested to be present in SI.4, PA-1, which was unlikely to result from the parallel dimer, could form PAA, APP and APA. If we consider a dimer addition to another dimer, this would give possible structures PPP, PAP1 and PAP2.

	**6-31G***		**6-31+G***		
**Structure**	**ZPE**	**Gibbs (300K)**	**ZPE**	**Gibbs (300K)**	**opt**
PPP	**0**	**0**			**0**
PAA	**16.8**	**28.6**	**0**	**0**	**12.9**
APP	**20.6**	**17.5**			
AAP	**29.4**	**34.7**			
PAP1	**30.3**	**35.3**	**6.4**	**4.5**	**19.0**
PPA	**38.5**	**44.1**	**30.7**	**26.4**	**45.9**
AAA1	**49.0**	**52.3**	**28.9**	**16.0**	**46.7**
PAP2	**50.4**	**42.0**			
APA	**58.8**	**50.0**	**37.0**	**14.3**	**56.6**
AAA2	**98.8**	**86.2**			

Table SI.1: Relative energies of the tetramer, both zero-point energy corrected (ZPE) and including Gibbs free energies at 300K for both basis sets. The right column designated "opt" is the optimization energy obtained, without ZPE or other corrections. All energies are in kJ/mol.

The IR spectra of the ten calculated structures are compared with the experimental IR spectrum by taking into account a known redshift above 1600 cm^{-1} resulting from the lower 6-31G* basis set. Based on the amide I region there is no structure that can be discarded: All structures show two distinct peaks although there are some differences in the distribution of the intensity in the peptide C=O peak. The amide II however allows us to exclude PAP1, which only shows a single peak. The fingerprint region of the IR spectrum is not conclusive either.

However, based on the general lay-out of the spectra, the energetics and the similarities with the spectra of the dimer and trimer, we conclude that the structure of the tetramer also adopts a beta-sheet structure in which all peptides are stacked on top of each other.

Concluding: The size of the tetramer made us decide to only calculate probable structures, i.e. intermolecular hydrogen bonded stacked peptides forming beta-sheet conformers. All beta-sheet structures show good agreement with the experiment. The energetics however showed that, as was observed for the dimer and the trimer, the all parallel structure was the lowest energy structure. The formation of an all parallel tetramer is expected as an extra monomer conformer is directly added to the observed trimer/dimer conformers. This is energetically favorable as only a C5 intramolecular hydrogen bond has to be broken and replaced by an intermolecular hydrogen bond.

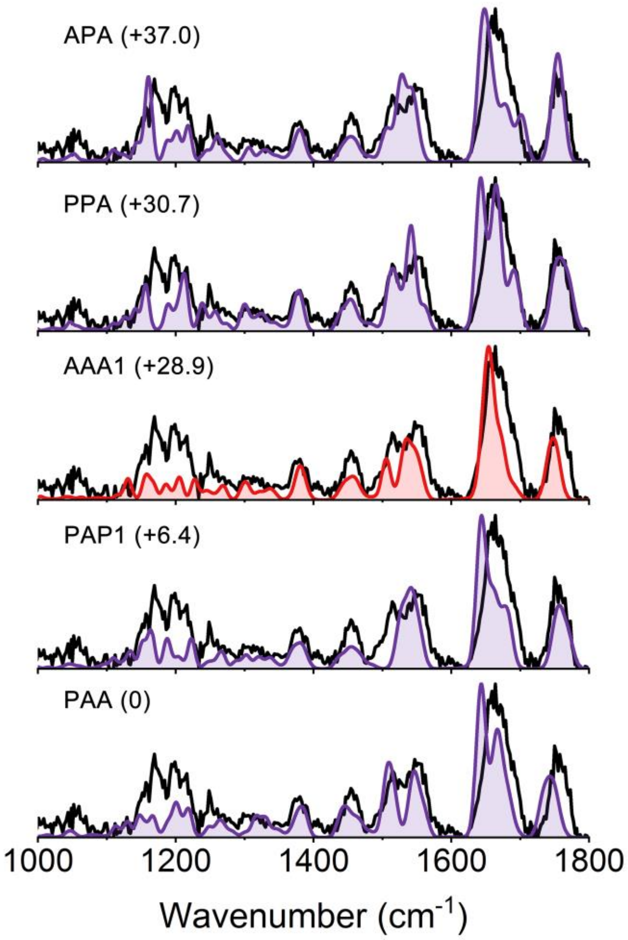

Figure SI.6C: Infrared spectra of five calculated beta sheet stacked tetramers in color, at the B3LYP-D3/6-31+G* level of theory, compared to the experimental spectrum in black. In the inset their respective structures are drawn, with red hydrogen bonds indicating an anti-parallel beta sheet and blue a parallel one. The names of the structures indicate which hydrogen bond types are present (P=parallel, A=anti-parallel).

7. Fourier Transform-Infrared data

Solid FT-IR spectroscopy was performed on Ac-Ala-Ala-OBn. The freeze-dried solid sample bought from BioMatik was used without any further treatment and was pressed in a KBr pellet in an approximate 1:600 ratio. The infrared spectra were measured in a Bruker Vertex 80V FT-IR spectrometer. Fig.SI.7 shows the FT-IR spectrum from 1000 to 1800 cm^{-1}. The amide I, sensitive to the secondary structure of the peptide, shows a strong peak at 1629 cm^{-1}, which is indicative of beta-sheet character, and a very weak feature at 1694 cm^{-1}. This last peak is indicative of anti-parallel structures, but points to parallel structures when (almost) absent, such as in this case.[8]

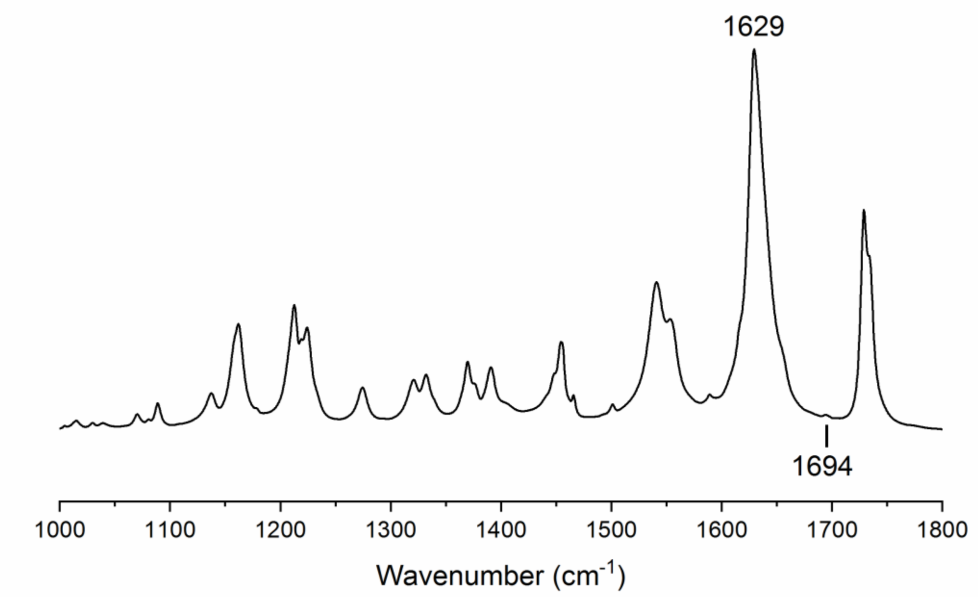

Figure SI.7A: Absorbance spectrum obtained in a FT-IR spectrometer. The peaks in the amide I region are indicated.

The peak positions of the amide I region were fitted (peak 1: 1640-1685, width 10<x<40; peak 2: 1690-1705, width 10<x<30) using two peaks, see Fig.SI.7B(a). The peaks were treated with a 5 point Savitzky-Golay smooth to determine the peak positions better. One peak is positioned around 1695 cm^{-1}, associated with non-hydrogen bonded C=O groups, and the other peak covering the rest of the band, indicating all hydrogen bonded C=O groups. A dotted line is put in the figure to guide the eye, from the maximum of the dimer peak to the maximum of the 9-mer peak. The resulting peak positions (maxima) of the hydrogen bonded peaks are

graphically shown in Fig.SI.7B(b). The overall red shift of this band with respect to the cluster size is clearly visible. The peak positions and the standard deviations derived from the fitting procedure are put in the table in Fig.SI.7B(c).

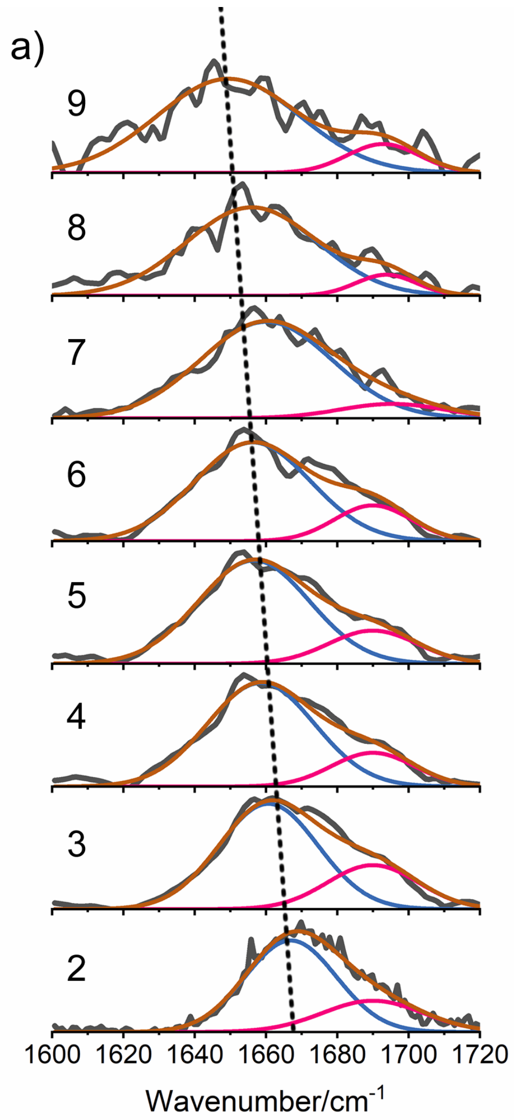

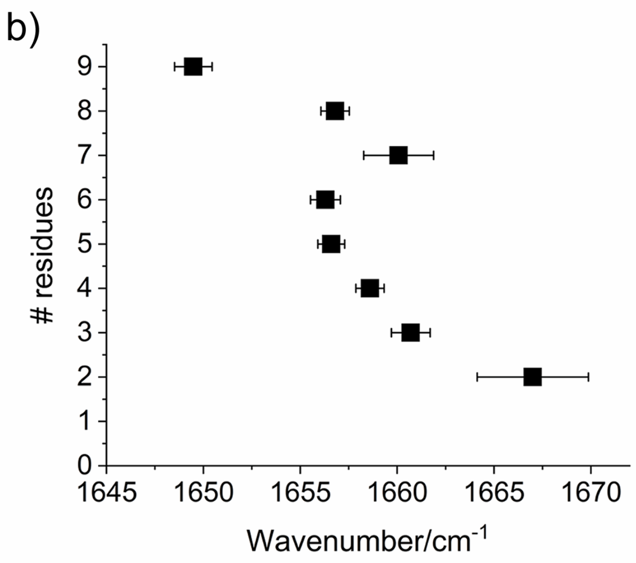

c)

Size of aggregate	Peak position (cm^{-1})
2	1667 (±2.87)
3	1660.7 (±1.00)
4	1658.6 (±0.73)
5	1656.6 (±0.69)
6	1656.3 (±0.77)
7	1658.1 (±2.85)
8	1656.1 (±0.73)
9	1649.5 (±0.97)

Figure SI.7B: Peak positions of the amide I band. a) Fitted peaks with in dark grey the experimental spectrum, blue the hydrogen bonded C=O peaks, in pink the free C=O groups and in orange the total fitted curve. The cluster size is indicated of the left of the spectra. The dotted line is linear between the peaks of the 2-mer and 9-mer.; b) Peak positions of the hydrogen bond fits, taken from the fitted spectra versus the size of the cluster, also tabled in c).

8. References in ESI:

1. A. M. Rijs and J. Oomens, *Top. Curr. Chem.*, 2015, **364**, 1-42.
2. D. Oepts, A. F. G. van der Meer and P. W. van Amersfoort, *Infrared Phys. Technol.*, 1995, **36**, 297-308.
3. D. A. Case, T. A. Darden, T. E. Cheatham III, C. L. Simmerlin, J. Wang, R. E. Duke, R. Luo, R. C. Walker, W. Zhang, K. M. Merz, B. Roberts, S. Hayik, A. Roitberg, G. Seabra, J. Swails, A. W. Götz, I. Kolossváry, K. F. Wong, F. Paesani, J. Vanicek, R. M. Wolf, J. Liu, X. Wu, S. R. Brozell, T. Steinbrecher, H. Gohlke, Q. Cai, X. Ye, J. Wang, M.-J. Hsieh, G. Cui, D. R. Roe, D. H. Mathews, M. G. Seetin, R. Salomon-Ferer, C. Sagui, V. Babin, T. Luchko, S. Gusarov, A. Kovalenko and P. A. Kollman, *AMBER 12*, 2012.
4. S. Grimme, J. Antony, S. Ehrlich and H. Krieg, *J. Chem. Phys.*, 2010, **132**.
5. M. J. Frisch, G. W. Trucks, H. B. Schlegel, G. E. Scuseria, M. A. Robb, J. R. Cheeseman, G. Scalmani, V. Barone, G. A. Petersson, H. Nakatsuji, X. Li, M. Caricato, A. Marenich, J. Bloino, B. G. Janesko, R. Gomperts, B. Mennucci, H. P. Hratchian, J. V. Ortiz, A. F. Izmaylov, J. L. Sonnenberg, D. Williams-Young, F. Ding, F. Lipparini, F. Egidi, J. Goings, B. Peng, A. Petrone, T. Henderson, D. Ranasinghe, V. G. Zakrzewski, J. Gao, N. Rega, G. Zheng, W. Liang, M. Hada, M. Ehara, K. Toyota, R. Fukuda, J. Hasegawa, M. Ishida, T. Nakajima, Y. Honda, O. Kitao, H. Nakai, T. Vreven, K. Throssell, J. A. J. Montgomery, J. E. Peralta, F. Ogliaro, M. Bearpark, J. J. Heyd, E. Brothers, K. N. Kudin, V. N. Staroverov, T. Keith, R. Kobayashi, J. Normand, K. Raghavachari, A. Rendell, J. C. Burant, S. S. Iyengar, J. Tomasi, M. Cossi, J. M. Millam, M. Klene, C. Adamo, R. Cammi, J. W. Ochterski, R. L. Martin, K. Morokuma, O. Farkas, J. B. Foresman and D. J. Fox, *Gaussian 09, Revision E.01*, 2016.
6. S. Bakels, E. M. Meijer, M. Greuell, S. B. A. Porskamp, G. Rouwhorst, J. Mahé, M. P. Gaigeot and A. M. Rijs, *Faraday Discuss.*, 2019, DOI: 10.1039/C8FD00208H.
7. E. Gloaguen, B. de Courcy, J. P. Piquemal, J. Pilme, O. Parisel, R. Pollet, H. S. Biswal, F. Piuzzi, B. Tardivel, M. Broquier and M. Mons, *J. Am. Chem. Soc.*, 2010, **132**, 11860-11863.
8. A. Barth, *Biochim. Biophys. Acta, Bioenerg.*, 2007, **1767**, 1073-1101.
